# Early endothelial progenitor cells and vascular stiffness in psoriasis and psoriatic arthritis

**DOI:** 10.1186/s40001-018-0352-7

**Published:** 2018-11-09

**Authors:** D. Patschan, N. Sugiarto, E. Henze, R. Mößner, J. Mohr, G. A. Müller, S. Patschan

**Affiliations:** 1 Department of Medicine I, Cardiology-Angiology-Nephrology, Klinikum Brandenburg, Medizinische Hochschule Brandenburg, Brandenburg, Germany; 2 Clinic of Nephrology and Rheumatology, University Hospital Göttingen, Göttingen, Germany; 3 Department of Dermatology and Venerology, University Hospital Göttingen, Göttingen, Germany

**Keywords:** eEPCs, Ps, PsA, CVR, Pulse wave velocity

## Abstract

**Background:**

Both psoriasis (Ps) and psoriasis arthritis (PsA) have been associated with increased cardiovascular risk. Also, both are characterized by increased neovascularization. Endothelial progenitor cells (EPCs) have been implicated in promoting vascular repair in ischemic diseases. The aim of the study was to correlate the EPC system with CV risk factors and with parameters of vascular stiffness in Ps and PsA.

**Methods:**

Twenty-six healthy subjects, 30 patients with Ps, and 31 patients PsA were included in the study. eEPC regeneration was evaluated by a colony-forming assay, circulating eEPCs were measured by cytometric analysis. For vascular analysis, all subjects underwent quantification of pulse wave velocity (PWV) and augmentation index (AIX).

**Results:**

Patients were categorized upon the duration of disease, severity of skin involvement (PASI-Ps), individual pain as reflected by the VAS (PsA), CRP values, and history of treatment with one or more biologicals. Regarding the eEPC system, no significant differences were observed between the respective categories. Correlation analyses between parameters of vascular stiffness (PWV and AIX) and patterns of colony formation/circulating eEPCs did not show any correlation at all.

**Conclusion:**

Parameters of vascular stiffness are not significantly deteriorated in Ps/PsA. Thus, pulse wave analysis may not be suitable for CVR assessment in certain autoimmune-mediated diseases. Regenerative activity of the eEPC system/circulating eEPC numbers are not altered in Ps/PsA. One may conclude that malfunctions of the eEPC are not substantially involved in perpetuating the micro-/macrovascular alterations in Ps/PsA.

## Introduction

The reported worldwide prevalence of psoriasis (Ps) is approximately 5% [1]. In about 25% of all Ps patients, morbidity is being aggravated by psoriasis arthritis (PsA). In most cases, PsA becomes manifest several years after the onset of Ps, in fewer patients arthritis coincides with Ps or even precedes cutaneous lesions years in advance [2]. Both Ps and PsA share common characteristics. On the one hand, many patients require systemic immunosuppressive therapy to control disease activity. On the other hand, the balance between de novo generation and decomposition of certain types of tissue is significantly deteriorated in both diseases: psoriatic skin lesions result from local hyperproliferation of keratinocytes and PsA-associated joint damage partly ensues from periarticular neoformation of bone and connective tissue [3, 4]. Tissue proliferation is typically associated with stimulated neovascularization under both physiological and pathological conditions (e.g., wound healing, tumor growth, chronic synovial inflammation and proliferation in rheumatoid arthritis). Another significant problem that may arise in Ps and PsA is increased cardiovascular risk (CVR) [5]. Higher insulin resistance and endothelial cell dysfunction have been shown to occur in Ps/PsA, thus significantly elevating CVR [6]. In this context, anti-TNF-alpha therapy has been shown to improve aortic stiffness in PsA patients which indicates favorable effects on CVR [7]. Overall, the vascular homeostasis is significantly impaired in Ps/PsA, partly as a result of pathological processes that occur in the skin and around joints and also due to systemic abnormalities that cause or are associated with increased CVR (e.g., systemic inflammation, treatment with immunosuppressive drugs).

Investigations performed in recent years identified endothelial progenitor cells (EPCs) as an essential regulatory element of the vascular system. The cells have initially been described by Asahara and colleagues [8] in the late 1990s, and since then a great effort has been made to further characterize EPCs. Very first conceptual ideas suggested the cells to be capable of replacing damaged mature endothelial cells within the (micro)vasculature [8, 9]. Meanwhile, it has come to attention that such direct vascular repair is most likely exclusively mediated by so-called late EPCs [10, 11]. Early EPCs (eEPCs), in contrast, predominantly act by indirect mechanisms including the release of paracrine substances and of proangiogenic microparticles [12, 13]. Therefore, eEPCs cannot reliably be defined as progenitors of endothelial cells but as proangiogenic hematopoietic cells with endothelial properties [14–16]. However, numerous studies revealed anti-ischemic effects of eEPCs under diverse experimental conditions [8, 15, 17, 18]. Also, alterations of the eEPC system have been identified in inflammatory and non-inflammatory vascular diseases such as ANCA (anti-neutrophil cytoplasmic antibodies)-associated vasculitis, and ischemic heart disease [19–22]. These data suggested that numbers of circulating eEPCs/eEPC regenerative activity is either affected by the disease and/or that functional alterations of the cells may be involved in perpetuating the vascular malfunction per se.

There are at least two reasons why we suspect to identify abnormalities of the eEPC system in Ps and PsA: (I) the chronic inflammatory process, accompanied by neovascularization in the skin (Ps) and around joints and bone (PsA) and (II) the higher CVR in both conditions. We intended to correlate the eEPC system with CVR risk factors and particularly with parameters of vascular stiffness.

## Methods

### Patients

Patients with psoriasis (Ps) and psoriasis arthritis (PsA) were recruited from the Clinic of Nephrology and Rheumatology and from the Clinic of Dermatology of the University Hospital Göttingen (Germany) over a period of 12 months. The study protocol was approved by the Institutional Review Board, and all patients provided written informed consent (name of the ethics committee: Medical Ethics Committee of the University Hospital of Göttingen—Grant number: 17/02/08).

All patients were either diagnosed with Ps by a local dermatologist or fulfilled the CASPAR (ClASsification criteria for Psoriatic Arthritis) criteria for PsA [23]. Disease activity was scored either using the Psoriasis Area Severity Index (PASI-Ps) or by evaluating the individual pain level (visual analog scale—VAS) and the C-reactive protein in PsA.

Depending on the severity of the disease, patients either underwent topical treatment regimens and/or received antiinflammatory/immunosuppressive protocols including one or more of the following substances: non-steroidal antiinflammatory drugs (NSAID); glucocorticoids, methotrexate; azathioprine; cyclosporine; sulfasalazine; leflunomide; chloroquine/hydroxychloroquine; adalimumab; certolizumab pegol; etanercept; golimumab; infliximab; ustekinumab. Healthy, age- and sex-matched individuals served as controls.

### Colony-forming unit assay and circulating eEPCs

The methods for culturing eEPCs and for cytometric analysis have extensively been published previously [20–22, 24]. However, the methods shall be described once again. Colony-forming units [CFU] assay: the assay was performed using the EndoCult Liquid Medium Kit^®^ (StemCell Technologies, Vancouver, BC, Canada) as per the manufacturer’s protocol. MNCs (mononuclear cells) were resuspended in complete EndoCult medium and seeded at 5 × 10^6^ cells/well on fibronectin-coated tissue culture plates (BD Biosciences, Rockville, MD, USA). After 48 h, wells were washed with media and non-adherent cells were collected. Non-adherent cells were plated in their existing media at 10^6^ cells/well in 24-well fibronectin-coated tissue culture plates for 3 days. Only colonies with at least 20 cells, containing rounded cells in the middle and elongated cells at the periphery, were considered as CFU-EC colonies. The numbers of colonies (colonies/well) appearing after this period were counted. At least two members of the laboratory staff evaluated the numbers of CFU-ECs. They were blinded for the diagnosis and status of the investigated patients/controls. The phenotype of cells within the colonies was determined more in detail. For this purpose, cells were characterized by the uptake of DiI-labeled acetylated low-density lipoprotein (acLDL) (Invitrogen, Carlsbad, CA, USA) and binding of FITC-labeled UE (*Ulex europaeus*) lectin (Sigma Diagnostics, St. Louis, MO). Cells were first incubated with 10 μg/ml DiI-ac-LDL at 37 °C for 1 h and later fixed with 2% formaldehyde for 10 min, followed by incubation with UE lectin at 37 °C for 1 h. The number of Dil-acLDL^+^/UE lectin^+^ cells was counted by laser scanning microscopy using an inverted fluorescence microscope IX-71 (Olympus Deutschland GmbH, Hamburg, Germany) equipped with the appropriate excitation and emission filters (AHF Analysentechnik, Tuebingen, Germany). Flow cytometry: for performing flow cytometry, mononuclear cells (MNCs) were isolated by density gradient centrifugation using Histopaque-1077 solution (Sigma Diagnostics, St. Louis, MO) from ≈ 7.5 ml of heparinized peripheral blood. Cells were primarily incubated for 1 h on ice with one or more of the following antibodies: rabbit anti-CD133 (ab16518-Abcam, Cambridge, UK), mouse anti-human VEGFR2 (KDR—Kinase insert Domain Receptor, directly conjugated-FAB 3571F—R&D Systems, Minneapolis, MN, USA), followed by secondary incubation with PE-conjugated goat anti-rabbit Fab (VEGFR, 111-116-144-Jackson Immunoresearch, Baltimore, PA, USA) for 30 min on ice, respectively. After incubation, cells were washed with PBS–BSA 1% (w/v). Data were acquired using a FACScalibur cytometer (Becton–Dickinson, Heidelberg, Germany) equipped with a 488-nm argon laser and a 635-nm red diode laser and analyzed using CellQuest software (Becton–Dickinson, San Jose, CA, USA). The setup of FACScalibur was performed according to the manufacturer’s instructions using unstained and single-antibody-stained cells. Specificity of staining was controlled by incubation with isotype-matched immunoglobulins. To quantify total peripheral endothelial cells, the numbers of KDR-positive cells, to quantify EPCs, the numbers of CD133/KDR double-positive cells within the myelomonocytic cell population were counted.

### Biochemical and hematological tests

Biochemical and hematological tests were performed in the Central Laboratories of our institution (University Medical Center Göttingen, Germany) adherent to the local standards.

### Pulse wave analysis and vascular augmentation index (AIX)

Assessment of pulse wave characteristics (reflecting central aortic blood pressures) and derivation of the vascular augmentation index (describing the reflected pulse wave when the systolic blood pressure is reflected by narrowing peripheral arteries) were performed by applanation tonometry using the Sphygmocor device (Version 7.0, AtCorMedical, West Ryde, New South Wales, Australia). Patients were examined first in a sitting position after at least 5-min rest. Then brachial blood pressure (BP) of the arm was measured with a semiautomatic oscillometric device (Bosch + Sohn GmbH, Juningen, Germany) and patient characteristics (age, gender, height, and weight) and BP were entered to the software. Mean BP (MBP) was calculated from systolic (SBP) and diastolic blood pressures (DBP) using the formula: MDP = DBP + 0.4 × (SBP − DBP). MBP was subsequently used for PWV analysis. Radial artery pressure waves were sampled over 10 s applying gentle pressure with the tonometer to the artery. The software calculated an average radial artery waveform; the corresponding central aortic pulse pressure (PP) was then derived using a validated transfer function [25]. The augmentation pressure (AP) was calculated from the difference between the first peak of the outgoing pressure wave to maximal pressure during systole. AP corresponds to the reflected pressure wave during systole. The augmentation index was calculated as the ratio of augmentation to central pulse pressure (AIX = AP/PP × 100). To eliminate variations due to different heart rates, the recordings were corrected by the SphygmoCor software to 75 beats per minute.

Then pulse wave analysis was performed with the patient in a supine position after another 5-min rest. Pulse rate and the distance from the jugulum to the strongest impulse of the carotid and femoral arteries were entered into the recording system. Pulse wave velocity was calculated by the pulse transit time divided by the travel distance. The PWVcf was measured with the SphygmoCor device by recording ECG-simultaneous arterial waveforms at the carotid and femoral arteries. Measured values are expressed as meters per second (m/s) and corrected according to age- and sex-specific reference values (PWVpredicted).

Quality criteria included visually acceptable pulse wave recordings with variation in pulse height, diastole and pulse lengths of 5% or less and mean pulse heights of at least 80 mV. This is expressed as the quality index (%), which is calculated automatically by the SphygmoCor software. For the PWV measurements, the time difference between ECG signal and the signal from the recording site (carotid or femoral artery) was deemed appropriate if the standard deviation was 10% or less of the mean value [26].

### Statistical analysis

Statistical analysis was performed using GraphPad Prism software (version 5, GraphPad Software, San Diego, USA). All values are expressed as mean ± SEM. The means of two or more populations were analyzed by Mann–Whitney test. Correlation analysis was performed by Spearman rank correlation test. Differences between two groups were considered significant at a *p* value < 0.05; a positive correlation was considered at *r* = 1.

## Results

We first would like to mention that all the *p* values are summarized in Table 1.

**Table 1:** *p* values of all subcategory-related analyses

	*p* value
CFU-ECs	
Ps < vs. ≥ mean DOD	0.15
PsA < vs. ≥ mean DOD	0.72
Ps < vs. ≥ mean PASI	0.94
PsA < vs. ≥ mean VAS	0.84
Ps biological− vs. biological+	0.94
PsA biological− vs. biological+	0.16
Ps < vs. ≥ mean CRP	0.53
PsA < vs. ≥ mean CRP	0.87
CD133^+^/KDR^+^ cells (%)	
Ps < vs. ≥ mean DOD	0.23
PsA < vs. ≥ mean DOD	0.65
Ps < vs. ≥ mean PASI	0.66
PsA < vs. ≥ mean VAS	0.11
Ps biological− vs. biological+	0.68
PsA biological− vs. biological+	0.58
Ps < vs. ≥ mean CRP	0.65
PsA < vs. ≥ mean CRP	0.24
PWV (m/s)	
Ps < vs. ≥ mean DOD	0.34
PsA < vs. ≥ mean DOD	0.70
Ps < vs. ≥ mean PASI	0.83
PsA < vs. ≥ mean VAS	0.59
Ps biological− vs. biological+	0.51
PsA biological− vs. biological+	0.42
Ps < vs. ≥ mean CRP	0.34
PsA < vs. ≥ mean CRP	0.07
AIX	
Ps < vs. ≥ mean DOD	0.2
PsA < vs. ≥ mean DOD	0.74
Ps < vs. ≥ mean PASI	0.63
PsA < vs. ≥ mean VAS	0.29
Ps biological− vs. biological+	0.09
PsA biological− vs. biological+	0.40
Ps < vs. ≥ mean CRP	0.43
PsA < vs. ≥ mean CRP	0.91

Ps, psoriasis; PsA, psoriasis arthritis; DOD, duration of the disease

### Subjects

Thirty patients with psoriasis (Ps) and 31 patients with psoriatic arthritis (PsA) were included in the study. Twenty-six healthy subjects served as controls. The following parameters were evaluated: gender, mean age, duration of the disease (DOD), CRP levels, skin involvement as reflected by the Psoriasis Area Severity Index (PASI), individual pain level as reflected by the VAS, treatment with one or more biological agents in the past/present, prevalence of arterial hypertension, prevalence of smoking, prevalence of statin treatment, prevalence of diabetes mellitus, pulse wave velocity (PWV), augmentation index (AIX), and eEPC-related parameters (CFU-ECs and CD133^+^/KDR^+^ cells). The baseline characteristics of all included patients are summarized in Table 2.

**Table 2:** Patient’s baseline characteristics (f: female; m: male)

	Ps	PsA
Sex	f: 13; m: 17	f: 15; m: 16
Age (years as mean ± SEM)	49.0 ± 2.8	47.7 ± 2.0
Duration of disease (DOD—mean years ± SEM)	18.3 ± 2.7	13.0 ± 2.4
CRP (mg/dl—mean ± SEM)	3.7 ± 0.7	5.1 ± 1.4
PASI	10.2 ± 2.0	–
Pain index (VAS in mm)	–	47.1 ± 4.4
Treatment with Biological (%)	33.3	45.1
Arterial hypertension (%)	40.0	41.9
Smoking (%)	70.0	64.5
Statin treatment (%)	3.3	19.3
Diabetes mellitus (%)	10.0	16.1
PWV (m/s—mean ± SEM)	8.0 ± 0.4	7.4 ± 0.3
AIX (%—mean ± SEM)	21.6 ± 2.8	19.8 ± 2.6
CFU-ECs (mean ± SEM)	22.1 ± 3.3	24.2 ± 3.1
CD133^+^/KDR^+^ cells (%—mean ± SEM)	8.0 ± 0.6	9.5 ± 1.5

### Blood-derived eEPC colonies and circulating eEPCs

**Fig. 1:**
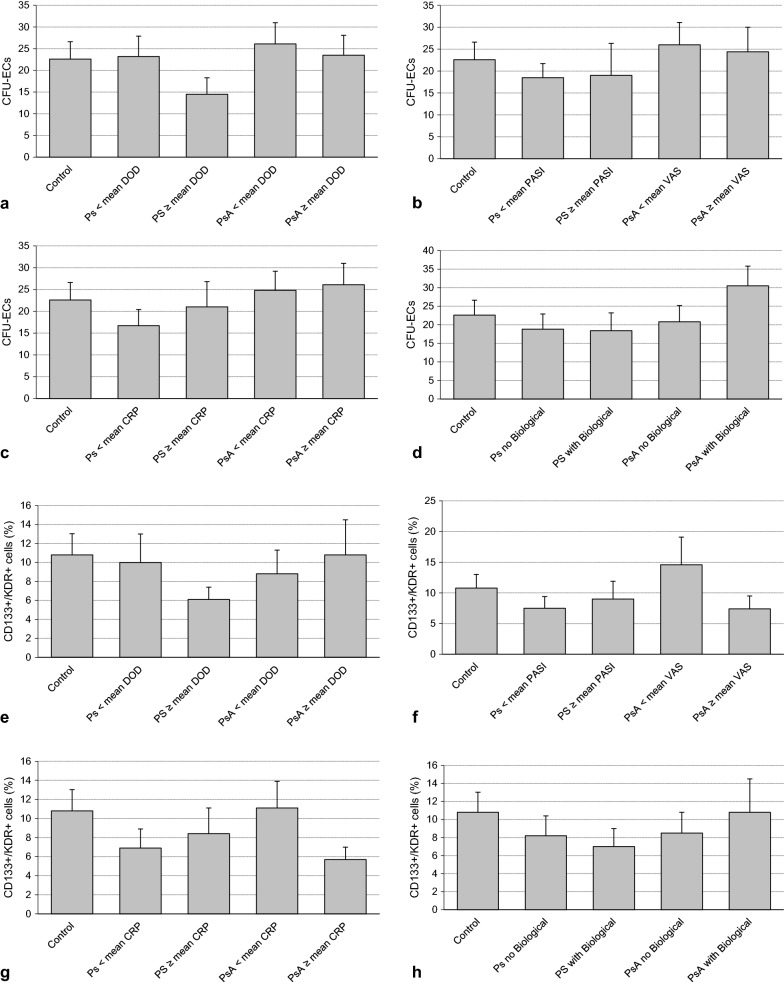
**a** CFU-ECs in relation to the mean DOD; **b** CFU-ECs in relation to PASI and VAS; **c** CFU-ECs in relation to CRP levels; **d** CFU-ECs in relation to biological treatment (yes vs. no); **e** circulating eEPCs (CD133^+^/KDR^+^ cells) in relation to the mean DOD; **f** circulating eEPCs (CD133^+^/KDR^+^ cells) in relation to PASI and VAS; **g** circulating eEPCs (CD133^+^/KDR^+^ cells) in relation to CRP levels; **h** circulating eEPCs (CD133^+^/KDR^+^ cells) in relation to biological treatment (yes vs. no)

Colony formation: the mean numbers of colonies were 22.6 ± 4.0 (controls); 22.1 ± 3.3 (Ps), and 24.2 ± 3.1 (PsA). Subgroup analyses revealed the following numbers of colonies in each category: below mean DOD—Ps 23.2 ± 4.7; PsA 26.1 ± 4.9; ≥ mean DOD—Ps 14.5 ± 3.8; PsA 23.5 ± 4.6; below mean CRP—Ps 16.7 ± 3.7; PsA 24.8 ± 4.4; ≥ mean CRP—Ps 21 ± 5.8; PsA 26.1 ± 4.9; below mean PASI (only Ps) 18.5 ± 3.2; ≥ mean PASI 19 ± 7.3; below mean VAS value (only PsA) 26 ± 5.1; ≥ mean VAS value 24.4 ± 5.6; no treatment with biological—Ps 18.8 ± 4.1; PsA 20.8 ± 4.4; treatment with biological—Ps 18.4 ± 4.8; PsA 30.5 ± 5.3; The differences between the respective categories (below/no vs. ≥/yes) were not statistically significant at all (Fig. 1).

Circulating eEPCs: the mean percentages of circulating eEPCs, as reflected by CD133^+^/KDR^+^ cells were 10.8 ± 2.2 (controls); 8.0 ± 0.6 (Ps) and 9.5 ± 1.5 (PsA). Subgroup analyses revealed the following percentages of circulating eEPCs in each category: below mean DOD—Ps 10.0 ± 3.0; PsA 8.8 ± 2.5; ≥ mean DOD—Ps 6.1 ± 1.3; PsA 10.8 ± 3.7; below mean CRP—Ps 6.9 ± 2.0; PsA 11.1 ± 2.8; ≥ mean CRP—Ps 8.4 ± 2.7; PsA 5.7 ± 1.3; below mean PASI (only Ps) 7.5 ± 1.9; ≥ mean PASI 9.0 ± 2.9; below mean VAS value (only PsA) 14.6 ± 4.5; ≥ mean VAS value 7.4 ± 2.1; no treatment with biological—Ps 8.5 ± 2.2; PsA 8.5 ± 2.3; treatment with biological—Ps 7 ± 2; PsA 10.8 ± 3.7; the differences between the respective categories (below/no vs. ≥/yes) (Fig. 1).

### Vascular stiffness

**Fig. 2:**
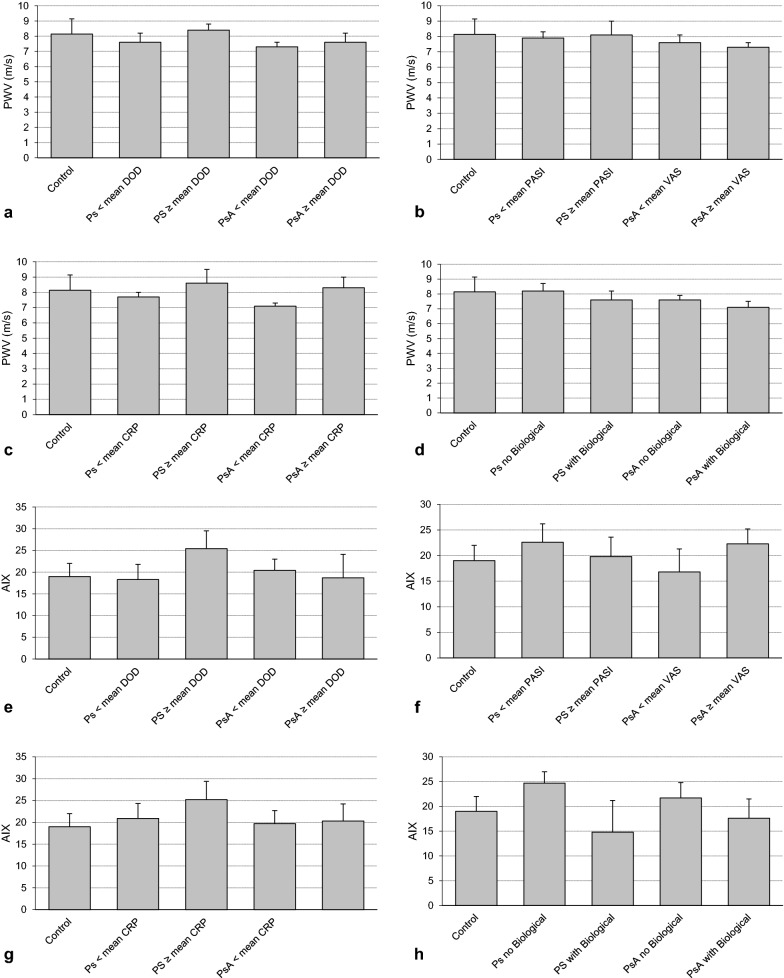
**a** PWV in relation to the mean DOD; **b** PWV in relation to PASI and VAS; **c** PWV in relation to CRP levels; **d** PWV in relation to biological treatment (yes vs. no); **e** AIX in relation to the mean DOD; **f** AIX in relation to PASI and VAS; **g** AIX in relation to CRP levels; **h** AIX in relation to biological treatment (yes vs. no)

Pulse wave velocity (PWV in m/s): the mean PWV were 8.1 ± 1.0 (controls); 8.0 ± 0.4 (Ps), and 7.4 ± 0.3 (PsA). Subgroup analyses revealed the following PWV in each category: below mean DOD—Ps 7.6 ± 0.6; PsA 7.3 ± 0.3; ≥ mean DOD—Ps 8.4 ± 0.4; PsA 7.6 ± 0.6; below mean CRP—Ps 7.7 ± 0.3; PsA 7.1 ± 0.2; ≥ mean CRP—Ps 8.6 ± 0.9; PsA 8.3 ± 0.7; below mean PASI (only Ps) 7.8 ± 0.4; ≥ mean PASI 8.5 ± 0.9; below mean VAS value (only PsA) 7.9 ± 0.4; ≥ mean VAS value 8.1 ± 0.9; no treatment with biological—Ps 8.2 ± 0.5; PsA 7.6 ± 0.3; treatment with biological—Ps 7.6 ± 0.6; PsA 7.1 ± 0.4; the differences between the respective categories (below/no vs. ≥/yes) were not statistically significant (Fig. 2).

Augmentation index (AIX): the mean PWV were 19.3 ± 3.0 (controls); 21.6 ± 2.8 (Ps), and 19.8 ± 2.6 (PsA). Subgroup analyses revealed the following AIX in each category: below mean DOD—Ps 18.3 ± 3.5; PsA 20.4 ± 2.6; ≥ mean DOD—Ps 25.4 ± 4.1; PsA 18.7 ± 5.4; below mean CRP—Ps 20.9 ± 3.4; PsA 19.7 ± 3.0; ≥ mean CRP—Ps 25.2 ± 4.2; PsA 20.3 ± 3.9; below mean PASI (only Ps) 22.6 ± 3.6; ≥ mean PASI 19.8 ± 3.8; below mean VAS value (only PsA) 16.8 ± 4.5; ≥ mean VAS value 22.3 ± 2.9; no treatment with biological—Ps 24.7 ± 2.3; PsA 21.7 ± 3.1; treatment with biological—Ps 14.8 ± 6.4; PsA 17.6 ± 3.9; the differences between the respective categories (below/no vs. ≥/yes) were not statistically significant at all (Fig. 2).

### Correlation analyses

**Fig. 3:**
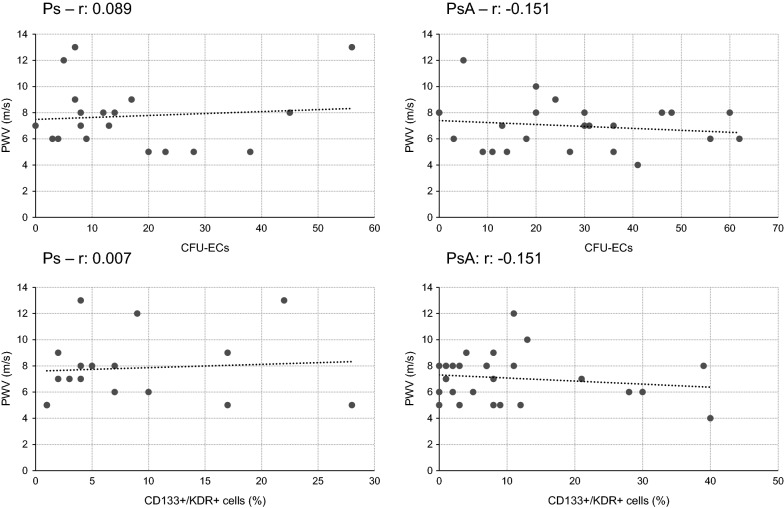
Correlation analysis between PWV and numbers of colonies/percentages of circulating eEPCs in Ps and PsA. None of the analyses showed any positive or negative correlation at all

**Fig. 4:**
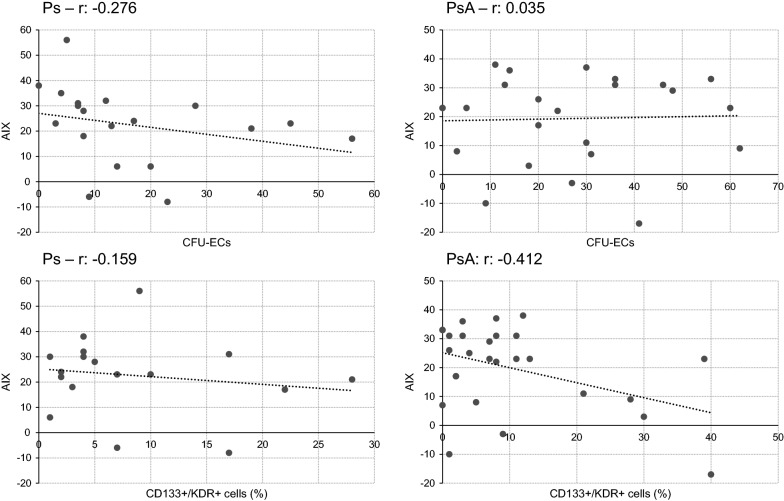
Correlation analysis between AIX and numbers of colonies/percentages of circulating eEPCs in Ps and PsA. Comparable to the previous analyses there were not any positive nor negative correlations at all

Finally, correlation analyses were applied to the following categories: PWV—mean numbers of colonies/circulating eEPCs in both Ps and PsA; AIX—mean numbers of colonies/circulating eEPCs in both Ps and PsA. This led to a total of eight separate correlation analyses. There were neither any positive nor negative correlations between any of the mentioned categories. The respective results including the correlation coefficients are summarized in Figs. 3 and 4.

## Discussion

The current study evaluated the eEPC system and parameters of vascular stiffness in Ps and PsA. Both diseases are characterized by deregulated proliferation of certain types of tissue (Ps—keratinocytes; PsA—connective tissue within/around joints) and by increased CVR [5]. The eEPC system, represented by a heterogenous populations of hematopoietic cells with proangiogenic properties [27], is critically involved in mediating vascular repair under pathological conditions such as ischemic heart, cerebrovascular, and renal disease [17, 19, 27, 28]. Also, certain inflammatory rheumatic disease has been shown to modulate percentages of circulating and proliferation of cultured (blood-derived) eEPCs, among those are rheumatoid arthritis (RA), systemic lupus erythematosus (RA), and Sjögren´s syndrome [22, 24, 29–34]. The cardiovascular risk is particularly increased in RA and SLE (systemic lupus erythematosus) [35]. One may speculate whether alterations of the eEPC system in these situations either reflect the endogenous failure of vascular repair mechanisms resulting in aggravated vascular damage (atherosclerosis) or evolve secondary, due to the inflammatory process and the drugs being used for disease control. Nevertheless, the current results are surprising since neither eEPC colonies nor percentages of circulating cells differed between controls and Ps/PsA. Comparable data have been presented by Ablin et al. [36]. Also, even if assigned to certain subcategories (e.g., duration of the disease, CRP values) differences remained non-significant. Two potential conclusions may be drawn. On the one hand, the eEPC system may not serve as ubiquitous surrogate marker of higher CVR in subjects with autoimmune-mediated inflammatory diseases. Second, and this particular aspect will also be addressed at the end of this section, subject numbers may be too low to identify significant differences between subcategories.

The second unexpected finding was the lack of any abnormalities in parameters of vascular stiffness. PsA patients with lower than average CRP concentrations displayed lower PWV values as compared to those with higher than average CRP levels. Nevertheless, the difference was only close to the level of significance, potentially attributable to the relatively small number of included subjects. Pulse wave velocity (PWV) has been proven as a useful predictor of cardiovascular events [37]. Meanwhile, a PWV of above 10 m/s has been accepted as a parameter of cardiovascular end-organ damage. Several studies analyzed parameters of vascular stiffness in Ps. Sunbul et al. found significantly higher AIX and PWV values in Ps as compared to controls [38]. Another study, however, failed to show any differences between patients and controls [39]. Regarding PsA, Costa et al. reported increased arterial stiffness even in the absence of known cardiovascular risk factors [40]. This observation was confirmed by another investigation [41]. In 2014, Brezinski and colleagues reviewed the literature on methods for evaluating endothelial function (including PWV and AIX analyses) in Ps and PsA, concluding that the majority of investigations showed higher than average PWV values in both diseases as compared to healthy subjects [42]. However, the methods for assessment of vascular function, especially for measuring PWV were not standardized across the included investigations. Thus, definite conclusions are impossible at the moment. We would, however, like to draw some attention to the study by Dowlatshahi and colleagues [39]. The aim of the ‘Rotterdam Study’ was to analyze the association between psoriasis and cardiovascular outcomes. A total of 262 Ps patients and 8.009 reference subjects were included and followed up for a mean of 11 years. The authors did not detect any difference in PWV between patients and controls, even after adjusting for age/gender. The reasons for such conflicting results are unknown, but one may raise the question whether procedures for quantification of PWV and AIX can reliably be used in chronic inflammatory diseases such as Ps, or PsA, or others. So far, the reliability of PWV analysis in certain diseases has only been studied to a limited extent. One investigation showed high reproducibility of PWV measurements in COPD [43]. Comparable investigations in rheumatic diseases are missing yet.

Finally, we would like to shortly discuss the weaknesses and the merit of the current study. The most important weakness is the comparably low number of subjects included. For instance, PWV was almost significantly higher in PsA patients with higher CRP values. One may speculate whether by increasing the number of analyses, this difference possibly became significant. The same conclusion must be drawn for other subcategories (e.g., AIX in Ps with vs. without biological treatment). Regarding PWV and the inflammatory activity, as reflected by the CRP, such an assumption was even plausible. Another weakness may be related to the enrollment site. All subjects were recruited from a university hospital in which they were followed up on a regular basis. Thus, CVR management may have been more efficient than in other regions of the country. However, this aspect remains completely speculative. The most significant merit, on the other hand, lies in the fact that procedures for quantification of PWV and AIX can obviously not reliably be used for CVR assessment in all types of chronic inflammatory diseases that have been reported to increase the cardiovascular risk.

## Conclusions

Taken together, we can summarize that neither the eEPC system nor parameters of vascular stiffness are compromised in Ps/PsA patients as compared to healthy controls. It remains questionable whether pulse wave analysis can reliably be performed in different rheumatic diseases characterized by increased CVR. Although higher CVR has been reported in both Ps and PsA, alterations of the eEPC system are not apparent/may not be useful to discriminate patients with average from those with increasing risk.
